# Exploring the role of monocyte chemoattractant protein-1 in fibroblast-like synovial cells in rheumatoid arthritis

**DOI:** 10.7717/peerj.11973

**Published:** 2021-08-11

**Authors:** Xiang Tong, Dongdong Yu, Li Yu, Weiqian Chen, Yanling Wen, Pengcheng Gu

**Affiliations:** 1 Department of Orthopedic Surgery, The First Affiliated Hospital, College of Medicine, Zhejiang University, Hangzhou, Zhejiang, China; 2 Operating Room, The First Affiliated Hospital, College of Medicine, Zhejiang University, Hangzhou, Zhejiang, China; 3 Department of Rheumatology, The First Affiliated Hospital, College of Medicine, Zhejiang University, Hangzhou, Zhejiang, China

**Keywords:** Rheumatoid arthritis, Fibroblast-like synoviocytes cells, Monocyte chemoattractant protein-1, Over-expression, Silence, Proliferation, Migration, Apoptosis, DAS28-MCP-1, Western blotting

## Abstract

**Background:**

Rheumatoid arthritis (RA) is a chronic systemic inflammatory disease with persistent synovitis. In the present study, the impact of monocyte chemoattractant protein-1 (MCP-1) was explored to determine methods for the diagnosis and treatment of RA.

**Methods:**

First, fibroblast-like synoviocytes (FLSs) were obtained from a collagen-induced rat RA model. Next, MCP-1-overexpression plasmid and small interfering RNA were transfected into human and rat FLSs. Cell Counting Kit-8 (CCK-8), Transwell migration and flow cytometry assays were used to analyze cell proliferation, migration and apoptosis of FLSs following MCP-1 transfections, respectively. Furthermore, western blotting was used to analyze the expression levels of p-P38, p-PI3K, PI3K, CD31, VEGF, TNF-α and IL-β in FLSs following MCP-1 transfection. In addition, reverse transcription-quantitative PCR and ELISAs were used to analyze the expression levels of C-reactive protein (CRP), estrogen receptor, MCP-1 and pentraxin-3 in patients with clinical RA, followed by correlation analysis of clinical data. Finally, expression validation, diagnostic and protein-protein interaction (PPI) network analysis of MCP-1 were performed.

**Results:**

MCP-1 promoted FLS proliferation and migration, and affected the apoptosis of FLSs. In addition, the expression levels of p-P38, p-PI3K, PI3K, CD31, VEGF, TNF-α and IL-β were also affected by MCP-1. In patients with clinical RA, the expression level of MCP-1 was increased. Moreover, CRP expression level was significantly up-regulated in RA. Clinically, MCP-1 was strongly correlated with tender joint count, swollen joint count, visual analog scale for general health and disease activity score 28 (DAS28)-MCP-1, and was moderately correlated with DAS28 and DAS28-CRP. PPI analysis showed that MCP-1 mainly interacted with other inflammatory cytokines.

**Conclusion:**

In conclusion, MCP-1 may play a significant regulatory role in RA, and could be used as a measurement index of clinical RA activity.

## Background

Rheumatoid arthritis (RA) is a chronic systemic inflammatory disease accompanied by persistent synovitis (Scott, Wolfe & Huizinga, 2010). The main characteristics of RA are inflammation of infiltrating joints, synovial hyperplasia and pathologic alteration of fibroblast-like synoviocytes (FLSs), which can lead to cartilage and bone damage and disability (Hu et al., 2019; Li et al., 2019; Smolen, Aletaha & McInnes, 2016). It has been shown that the incidence of RA is high among women, smokers and individuals with family history (Wasserman, 2011). The common cure for RA is the use of disease-modifying antirheumatic drugs (Smolen et al., 2014). The earlier a patient with RA is diagnosed and treated, the more favorable the long term prognosis (Brown & Isaacs, 2014).

Synovial fibroblasts (FLSs), also known as B-type synovial cells, are the main cell type that constitutes the inner membrane structure of the synovium (Wilkinson et al., 1992). As the main component of the synovial intima, FLSs play a significant role in the progression of RA. The proliferative and anti-apoptotic ability of FLSs are enhanced in RA (Li et al., 2019). In RA, FLSs damage joints by producing cytokines, chemokines and matrix degradation molecules, as well as migrating and invading articular cartilage (Bustamante et al., 2017). Monocyte chemoattractant protein-1 (MCP-1), also known as CCL2, is a member of the CC-motif chemokine family, which plays a role in RA by binding to the C-C motif chemokine receptor 2 (Deshmane et al., 2009). The migration of chemokine-mediated inflammatory cells into the joints is one of the main characteristics of RA (Trzybulska et al., 2018). Previous studies have shown that the expression level of MCP-1 in RA serum was higher compared with that in normal serum (Koch et al., 1992; Trzybulska et al., 2018). In addition, MCP-1 plays a significant role in angiogenesis and tumor progression (Chen et al., 2016; Trzybulska et al., 2018). MCP-1 is also associated with the differentiation and maturation of osteoclasts, indicating a relationship between MCP-1 and bone remodeling (Morrison, Day & Nicholson, 2014).

The pathogenesis of RA is currently unclear. Therefore, it is important to determine the role of MCP-1 in RA, and combine existing known clinical features with MCP-1 to assess clinical arthritis activity, which could assist the early diagnosis of RA. In the present study, human and rat FLSs were transfected with MCP-1 overexpression plasmid and small interfering RNA (siRNA) targeting MCP-1 for further analysis. The present results showed that MCP-1 affected the proliferation, migration and apoptosis of FLSs. In addition, combined with clinical features, MCP-1 may be used as a measurement index for evaluating clinical arthritis activity.

## Material and Methods

### Construction of collagen induced RA rat model

The RA rat model was immunologically induced by bovine type II collagen. Forty-five rats were purchased from Zhejiang University, Hangzhou, China. Male Sprague-Dawley rats (age: 8 weeks) were feed adaptively for 1 week. Next, type II collagen was solubilized in 0.1 M acetic acid to a final concentration of 2 mg/ml and placed in a refrigerator at 4 °C overnight. The next day, type II collagen was mixed with an equal volume of incomplete Freund’s adjuvant and stirred on ice until completely fused as an antigen emulsion. Rats were anesthetized by intraperitoneal injection of 10% chloral hydrate (300 mg/kg). No animals exhibited signs of peritonitis following the administration of 10% chloral hydrate. Rats were then subcutaneously inoculated with 0.3 ml antigen emulsion on days 0 and 7. The rats in the normal control group were injected with an equal volume of normal saline.

### Isolation and identification of FLSs from the RA rat model

First, rats were anesthetized by an intraperitoneal injection of 10% chloral hydrate (300 mg/kg) and euthanatized by cervical vertebra dislocation before experiments. No animals exhibited signs of peritonitis following the administration of 10% chloral hydrate. Furthermore, no pain, suffering, disease or other symptoms occurred during the process of euthanasia. Next, the synovial tissue was collected and washed 3–5 times on ice with sterilized PBS. Third, the synovial tissue was sliced into one mm^3^ thick slices and placed into a Petri dish containing 0.2% type II collagenase and incubated at 37 °C. Next, the supernatant was collected every 60 min and centrifuged for 5 min (1000 rpm/min) to collect the cell pellets. Subsequently, cells were filtered through the 200-mesh stainless steel filter and inoculated in a culture flask at 37 °C. Finally, when the cells had reached 90% fusion, the cell passage was carried out. The cells passaged to the second generation were used for subsequent experiments.

Vimentin, a marker of FLSs (Zhang et al., 2018), was used for FLS identification. In the culture plate, the slides containing the cells were washed with PBS thrice (3 min/time), fixed with 4% paraformaldehyde for 15 min and washed with PBS. The slides were then permeated with 0.5% Triton X-100 at room temperature for 20 min. Slides were washed with PBS thrice (3 min/time) and blocked with goat serum at room temperature for 30 min. The slides were incubated with diluted primary antibody (vimentin antibody, bs-0756r, Bioss) and incubated overnight at 4 °C in a humid box. The slides were washed with PBS thrice (3 min/time) and incubated with the diluted secondary antibody at 37 °C for 1 h. Then, the slides were washed with PBS thrice (3 min/time). Finally, 3, 3′-diaminobenzidine (DAB) color processing and observation under a microscope were performed to capture the image. The cytoplasm of FLSs was brownish yellow due to the expression of vimentin.

All experimental procedures were approved by the Animal Ethics Committee of the First Affiliated Hospital of the College of Medicine, Zhejiang University (approval no.20201172) and carried out accordance with the Zhejiang Province Experimental Animal Management Regulations.

### Culture and transfection of FLSs

The human FLSs (purchased from Shanghai Xinyu Biotechnology Co., Ltd) and rat FLSs were cultured in complete medium in a thermostatic incubator at 37 °C with 5% CO_2_. The human MCP-1 and rat MCP-1-overexpression plasmids [constructed by the pcDNA3.1 (+) vector] were purchased from Beijing Bomade Genetic Technology Co., Ltd. In addition, chemosynthetic human siRNA and rat siRNA were obtained from Huzhou Hippo Biotechnology Co., Ltd. Empty vector and scrambled siRNA were used as the normal controls for overexpression and siRNA transfection, respectively.

Previous studies have demonstrated that MCP-1 could enhance the growth rate through a transfection experiment (Kuroda et al., 2005; Zhang et al., 2015). In the present study, human and rat FLSs were transfected with MCP-1-overexpression plasmid and siRNA for further analysis. One day before transfection, 5.0−8.0 × 10^4^ cells were inoculated with 2 ml serum-free medium into a 6-well plate. Transfection was performed when cell confluence reached 70–90%. First, 250 µl serum-free medium and 0.8 µg DNA or siRNA (final concentration, 100 nM) were mixed. Next, 250 µl serum-free medium and 2 µl Llipofectamine^®^ 3000 (Invitrogen; Thermo Fisher Scientific, Inc.) were also mixed. These two solutions were incubated at room temperature for 5 min. Subsequently, the two mixtures (DNA or siRNA+ Llipofectamine 3000) were mixed and incubated at room temperature for 20 min. The normal medium in the 6-well plate was replaced with 1.5 ml medium. A total of 500 µl of DNA/ Lipofectamine 3000 or siRNA/ Lipofectamine 3000 mixtures were added dropwise into the 6-well plate and shaken gently. After 4-6 h, serum-free medium was replaced with complete medium. The cell transfection efficiency was detected after 48 h.

### Detection of MCP-1 transfection efficiency

In the present study, reverse transcription-quantitative PCR (RT-qPCR) and immunofluorescence analysis were used to analyze the MCP-1 transfection efficiency at the mRNA and protein level, respectively. Cell samples from normal control, MCP-1-overexpression and MCP-1-silencing groups were obtained for RT-qPCR. Total RNA was extracted using a TRIzol^®^ kit (cat. no. 10296028; Invitrogen; Thermo Fisher Scientific, Inc.). Nucleic acid concentration analyzer was used to detect the concentration and purity of total RNA. SuperScript III RT reverse transcription kit (cat. no. 11752050; Thermo Fisher Scientific, Inc.) was used to synthesize the cDNA. SYBR^®^qPCR mix (cat. no. 4472920; Thermo Fisher Scientific, Inc.ABI-invitrogen) was used for the RT-qPCR. Each experiment was repeated for three times. Actin was used as the internal reference. The relative gene expression levels were calculated as fold-changes using the 2^−ΔΔCt^ method (Livak & Schmittgen, 2001).

It is found that MCP-1 is expressed in the cytoplasm in the immunofluorescence analysis (Kuroda et al., 2005; Lazar et al., 2010; Zhang et al., 2015). Therefore, immunofluorescence was used to detect the transfection efficiency in present study. For immunofluorescence analysis, before adding the fluorescent secondary antibody (Flow-cyt/IF, Sheena Selvey, diluted 200 times), the slides were soaked three times (3 min/time) with PBS Tween-20 (PBST). Diluted fluorescent secondary antibody was added to the slides and incubated in a humid box at 37 °C for 1 h. Slides were then washed with PBST for three times (3 min/time). Next, DAPI was added to the slides and incubated in the dark for 5 min for nuclear staining. The extra DAPI was washed away with PBST for 4 times (5 min/time). The slides were sealed with a sealing solution containing anti-fluorescence quencher and observed under a fluorescence microscope at a magnification of 400 X. Normal human and rat FLSs were used as the controls.

### Cell Counting Kit-8 (CCK-8) assay

The cell suspension was prepared and inoculated into the 96-well plate (100 µl per well; three replicate wells per condition) and cultured for 24 h to allow cells adhere to the wall. MCP-1-overexpression plasmid or siRNA was transfected into cells and incubated in complete medium for 48 h. CCK-8 solution (10 µl) was then added to the cells and incubated for 1 h. The absorbance was measured at 450 nm using a microplate reader. The absorbance value was directly proportional to the cell proliferative ability.

### Transwell migration assay

Prior to the experiment, the cells were starved in serum-free medium for 12 h. Following digestion with trypsin, the cells were centrifuged for 3 min (1,000 rpm/min) to discard the supernatant. Next, the cells were resuspended in PBS and centrifuged to discard the supernatant. The cells were resuspended in serum-free medium containing 0.1% BSA and counted. The serum-free medium containing 0.1% BSA was used to adjust the cell density to 1 × 10^5^ cells/ml. Complete medium (700 µl) and cell suspension (200 µl) were added to the 24-well plate and inside the Transwell chamber, respectively. Tweezers were used to carefully transfer the chamber to a 24-well plate, avoiding the formation of air bubbles. The 24-well plate was gently placed into the 37 °C cell incubator containing 5% CO_2_ for 24–48 h. After gently removing the medium from the upper chamber, the cells of the upper chamber were wiped removed with a PBS-soaked cotton swab. The bottom of the chamber was immersed in a 10% methanol solution to fix the cells for 30 s, and then transferred to pure water to wipe off the methanol solution. The bottom of the chamber was immersed in the crystal violet dye solution for 2 min and washed with pure water until the background was clear. The number of cells passing through the membrane was counted under a microscope at a magnification of 100 X.

### Detection of cell apoptosis by flow cytometry

The cell culture fluid was collected in a suitable centrifuge tube for later use. The adherent cells were washed with PBS. Trypsin without ethylenediaminetetraacetic acid was used to digest the cells. The previously collected cell culture fluid was used to disperse gently all the adherent cells. The cells were centrifuged for 5 min (1,000 rpm/min) to collect the cells and discard the supernatant. They were then resuspended with ∼1 ml PBS (pre-cooled at 4 °C) and centrifuged again to discard the supernatant. The mixture (4 ml 4X binding buffer and 12 ml deionized water) was used to resuspend the cells and adjust the cell concentration to 1–5 × 10^6^ cells/ml. The cell suspension (100 µl) and Annexin V-FITC (5 µl) was mixed in a 5-ml flow tube incubated at room temperature for 5 min in the dark. Next, 10 µl of 20 µg/ml propidium iodide (PI) solution and 400 µl of PBS were added for flow cytometry analysis.

### Western blotting

In our previous study, the cell lysis method was used to detect the expression levels of MCP-1 and VEGF by western blotting (Tong et al., 2020). Similarly, in a study by Du et al. (2016) the cells were lysed for western blotting to determine the expression levels of MCP-1 and VEGF. In the present study, western blotting was used to detect the expression levels of MCP-1, phosphorylated (p)-p38 mitogen-activated protein kinase (p38), p-PI3K, PI3K, CD31, VEGF, TNF-α and IL-β. Extracting solution of protein (cat. no. MDL91201; MDL) was used for protein extraction in the logarithmic growth phase cells. Bicinchoninic acid protein concentration determination kit (cat. no. MD913053; MDL) was used for the detection of protein concentration. Sodium dodecyl sulfate polyacrylamide gel electrophoresis prefabricated adhesive kit (cat. no. MD911919, MDL) was used for the isolated protein bands. The protein bands on the gel were transferred to a polyvinylidene fluoride membrane (ISEQ00010, EMD, Millipore) and incubated with the primary and subsequent secondary antibodies (MD912565, MDL, diluted 2000 times). Finally, the efficient chemiluminescence kit was used to visualize protein bands. Bands from three separate western blotting experiments were analyzed by Quantiscan software. Actin was used as an internal control for protein detection. Result was presented as target protein/internal reference, which was regarded as the relative expression of target protein. Unpaired t test was used for statistical significance analysis.

### Detection of MCP-1 expression level in clinical RA

In the present study, the diagnostic criteria of RA were in accordance with the American College of Rheumatology in 1987. The detailed inclusion criteria for patients with RA were as follows: (i) Patients presented with morning stiffness for at ≥1 h (≥6 weeks); (ii) patients presented with swelling in ≥3 joints (≥6 weeks); (iii) patients presented with swelling of the wrist, palm, finger or proximal interphalangeal joint (≥6 weeks); (iv) patients presented with symmetrical joint swelling (≥6 weeks); (v) patients presented with typical radiological changes of RA in the hand; (vi) patients presented with rheumatoid nodules under the skin; and (vii) patients had a positive rheumatoid factor (titer, >1:32). Patients presented with more than four of the above symptoms were diagnosed with typical RA. Patients complicated by hypertension, coronary atherosclerotic heart disease, diabetes, active infection, connective tissue disease and pregnancy were excluded. According to the above inclusion and exclusion criteria, 13 patients with RA and 13 healthy individuals were enrolled in the study. During the RNA extraction process, two inappropriate samples were discarded.

The whole blood samples of these individuals were obtained for RT-qPCR. Total RNA was extracted using a TRIzol^®^ kit (cat. no. 10296028; Invitrogen; Thermo Fisher Scientific, Inc.). A total of 0.25 mL liquid sample was added with 0.75 mL lysate RLS and then shaken vigorously for 30s, and incubated at 15−30 °C for 10min. 0.2 mL chloroform was added to each 0.75 mL RLS, violently shaken for 15 s and placed at room temperature for 5 min. After centrifuged for 10 min (1000 rpm/min) at 4 °C, the upper colorless aqueous phase was transferred to a new Ep tube. 1 times volume 70% ethanol was mixed and transferred to adsorption column RA. After centrifuging for 45s (12000 rpm/min), the waste solution was discard. 0.5 mL of protein-removing solution RE was added and centrifuged for 45s (12000 rpm/min). The waste solution was discarded. 0.5 mL of rinsing solution RW was added and centrifuged for 45s (12000 rpm/min). The waste solution was discarded (repeat once). In order to remove the rinsing solution as much as possible, mixture was centrifuged again for 2 min (13000 rpm/min). Finally, RNase free water was added for elution. Nucleic acid concentration analyzer was used to detect the concentration and purity of total RNA. SuperScript III RT reverse transcription kit (cat. no. 11752050; Thermo Fisher Scientific, Inc.) was used to synthesize the cDNA. SYBR^®^qPCR mix (cat. no. 4472920; Thermo Fisher Scientific, Inc.ABI-invitrogen) was used for the RT-qPCR. Each experiment was repeated for three times. Actin was used as the internal reference. The relative gene expression levels were calculated as fold-changes using the 2^−ΔΔCt^ method (Livak & Schmittgen, 2001). All primers used in the present study are shown in Table 1.

All experimental procedures were approved by the Clinical Research Ethics Committee of the First Affiliated Hospital of the College of Medicine of Zhejiang University (approval no. 2020861). Written informed consent was obtained from all patients.

### ELISAs

ELISA kits were used to detect the content of Estrogen receptor (ER), pentraxin-3 (PTX3), MCP-1 and C-reactive protein (CRP) in normal and AR serum samples, respectively. Briefly, samples and enzyme-labeled reagents were added to each well and incubated at 37 °C for 60 min. The washing buffer was added to each well and discarded after being motionless for 30 s (5 thrice). Color development reagent and stop solution were used for the dye (15 min) and stop reaction, respectively. Finally, the optical density value of each hole was measured at a wave length of 450 nm.

**Table 1:** Primer sequences used for the reverse transcription-quantitative PCR.

	**Primer name**	**Primer sequence (5′to 3′)**
Cell	Actin (human)-F(internal reference)	5′-GACAGGATGCAGAAGGAGATTACT-3′
	Actin (human)-R(internal reference)	5′-TGATCCACATCTGCTGGAAGGT-3′
	MCP-1 (human)-F	5′-GCTCAGCCAGATGCAATCAA-3′
	MCP-1 (human)-R	5′-ACAGATCTCCTTGGCCACAA-3′
	Actin (rat)-F(internal reference)	5′-CCAGCCTTCCTTCTTGGGTA-3′
	Actin (rat)-R(internal reference)	5′-CAATGCCTGGGTACATGGTG-3′
	MCP-1 (rat)-F	5′-CAGCCAACTCTCACTGAAGC-3′
	MCP-1 (rat)-R	5′-GTGAACAACAGGCCCAGAAG-3′
Clinical	Actin (human)-F(internal reference)	5′-CCAGCCTTCCTTCTTGGGTA-3′
	Actin (human)-R(internal reference)	5′-CAATGCCTGGGTACATGGTG-3′
	MCP-1 (human)-F	5′-CAGCCAACTCTCACTGAAGC-3′
	MCP-1 (human)-R	5′-GTGAACAACAGGCCCAGAAG-3′

**Notes.**

Cell RT-PCR was used for detection of MCP-1 expression level in transfected cells Clinical RT-PCR was used for detection of MCP-1 expression level in clinical RA patients

### Correlation analysis of clinical data

In the blood of RA and healthy individuals, tender joint count (TJC), swollen joint count (SJC), visual analog scale for gener al health (GH) and disease activity score 28 (DAS28) were calculated. The MCP-1 data was added to the DAS28 calculation formula as follows (Liou et al., 2013; Prevoo et al., 1995): DAS28-MCP-1 = 0.56x $\sqrt{TJC}$ +0.28x $\sqrt{SJC}$+0.39xln(MCP-1) +0.014x(GH). The R 4.0.2 package was used to analyze the correlation among the expression of TJC, SJC, GH and MCP-1 in the sample. Shapiro–Wilk Test was used to verify whether each group of values was normally distributed (*P* > 0.05 means normally distributed). Pearson correlation coefficient and Spearman’s correlation coefficient were used for normally and non-normally distributed data, respectively.

### Expression validation, diagnostic and protein-protein interaction (PPI) network analysis of MCP-1

Expression validation, diagnostic and protein-protein interaction (PPI) network analysis of MCP-1 was performed using the GSE103578 and GSE128813 datasets, which were obtained from the GEO database (Edgar, Domrachev & Lash, 2002). The Search Tool for the Retrieval of Interacting Genes/Proteins (STRING) database (https://string-db.org/) was used to construct the PPI network. Subsequently, Cytoscape software (http://www.cytoscape.org) was used for the visualization of the PPI network.

### Statistical analysis

All statistical analysis was performed using GraphPad Prism (GraphPad Software, Inc.). *P* < 0.05 was considered statistically significant. One-way ANOVAs were used to analyze the RT-qPCR results. Data are presented as the mean ± SD.

## Results

### Identification of rat FLSs and detection of MCP-1 transfection efficiency

**Figure 1:**
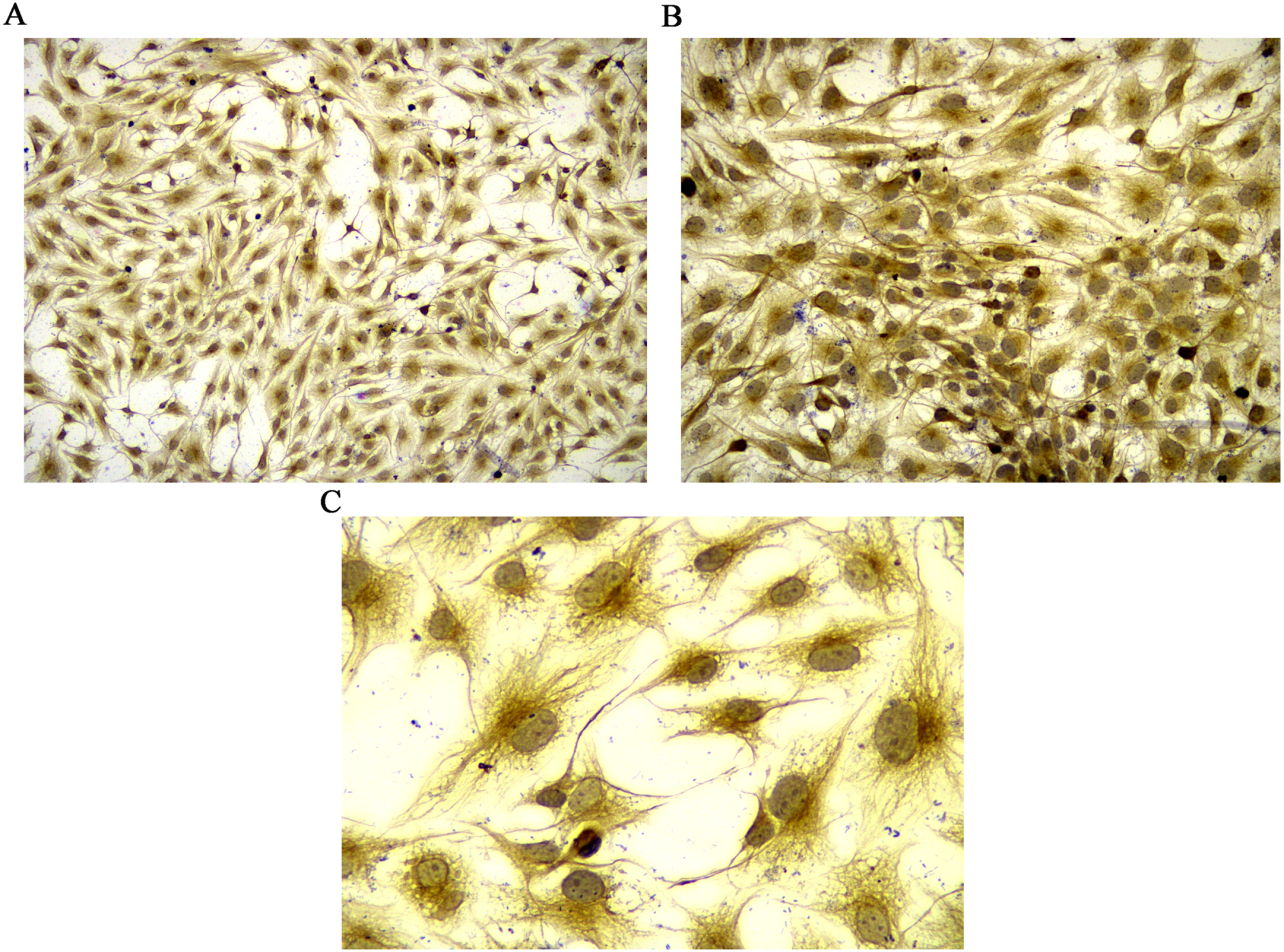
Identification of rat FLSs. (A) Observation under 100 X microscope. (B) Observation under 200 X microscope. (C) Observation under 400 X microscope. FLSs, fibroblast-like synoviocytes.

Vimentin was used to identify isolated rat FLSs, which was shown in Fig. 1. The mRNA expression level of MCP-1 were analyzed following 48 h of transfection. Following overexpression and silencing, the expression level of MCP-1 in both human and rat FLSs were significantly increase and decreased, respectively (Figs. S1A and S1B). Immunofluorescence was used to further validate the transfection efficiency of MCP-1 at the protein level. Under a fluorescence microscope, the cell nucleus was marked with blue fluorescence. The MCP-1 protein was marked with green fluorescence. Following the overexpression and silencing of MCP-1, the expression level of MCP-1 in human FLSs exhibited an increased and decreased trend, respectively, compared with normal human FLSs (Figs. S1C and S1E). However, the trend was not obvious enough, which may be associated with the state of the cells. Rat FLSs also showed a similar trend after MCP-1 transfection (Figs. S1F and S1H). This indicated that the transfection was successful and could be used for subsequent experiments.

### MCP-1 promotes FLS proliferation and migration

**Figure 2:**
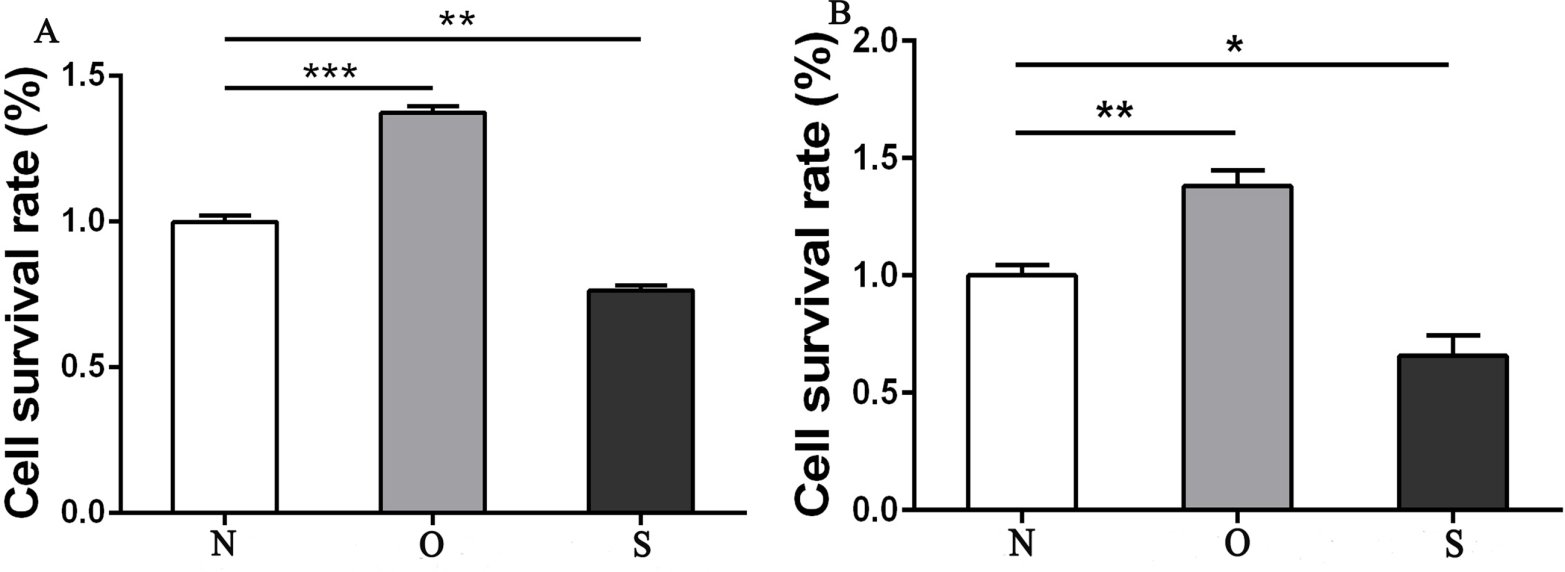
CCK8 assay of FLSs following MCP-1 transfection. (A) Survival rate of human FLSs following transfection. (B) Survival rate of rat FLSs following transfection. N, O and S represents the normal control group, MCP-1-overexpression group and MCP-1-silencing group, respectively. * *P* < 0.05, ** *P* < 0.01 and *** *P* < 0.001. FLSs, fibroblast-like synoviocytes; MCP-1, monocyte chemoattractant protein-1. Each experiment was repeated for three times.

**Figure 3:**
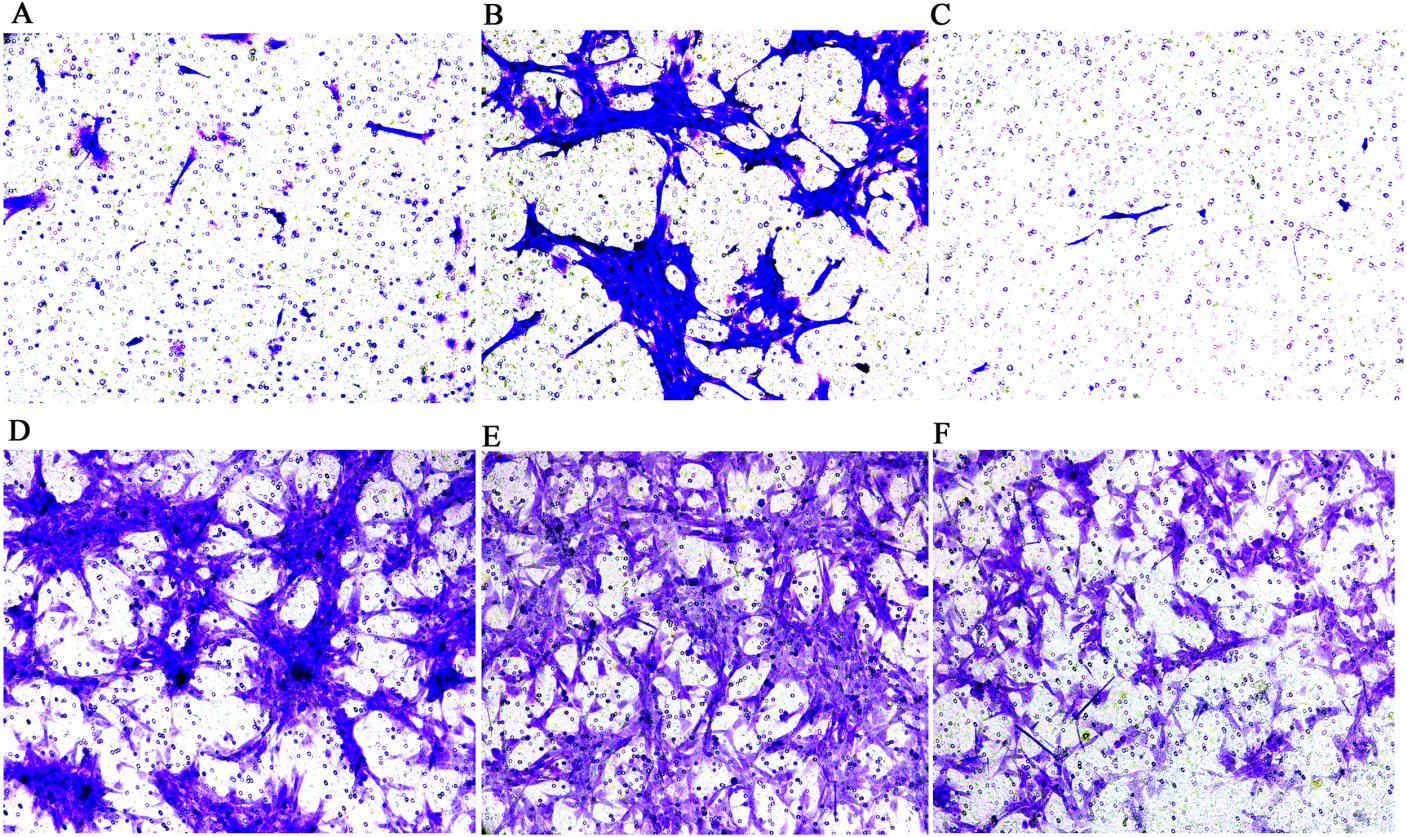
Transwell migration assay of FLSs following MCP-1 transfection. (A) Normal human FLSs. (B) Overexpression of MCP-1 in human FLSs. (C) MCP-1-silencing in human FLSs. (D) Normal rat FLSs. (E) Overexpression of MCP-1 in rat FLSs. (F) MCP-1-silencing in rat FLSs. Magnification, 100 X. FLSs, fibroblast-like synoviocytes; MCP-1, monocyte chemoattractant protein-1.

The results of the CCK-8 analysis showed that following MCP-1-overexpression, the cell viability of FLSs in humans and rats was enhanced, which indicate that MCP-1 promoted cell proliferation. In the MCP-1-silencing group, the viability of FLSs in humans and rats was weakened, which inhibited cell proliferation (Fig. 2). Transwell migration assay was used to detect the migratory ability of FLSs following MCP-1 transfection. MCP-1-overexpression promoted the migratory ability of FLSs, as compared with the control group. Nevertheless, the migratory ability of FLSs was weakened following MCP-1-silencing (Figs. 3A–3F).

### MCP-1 affects FLS apoptosis

**Figure 4:**
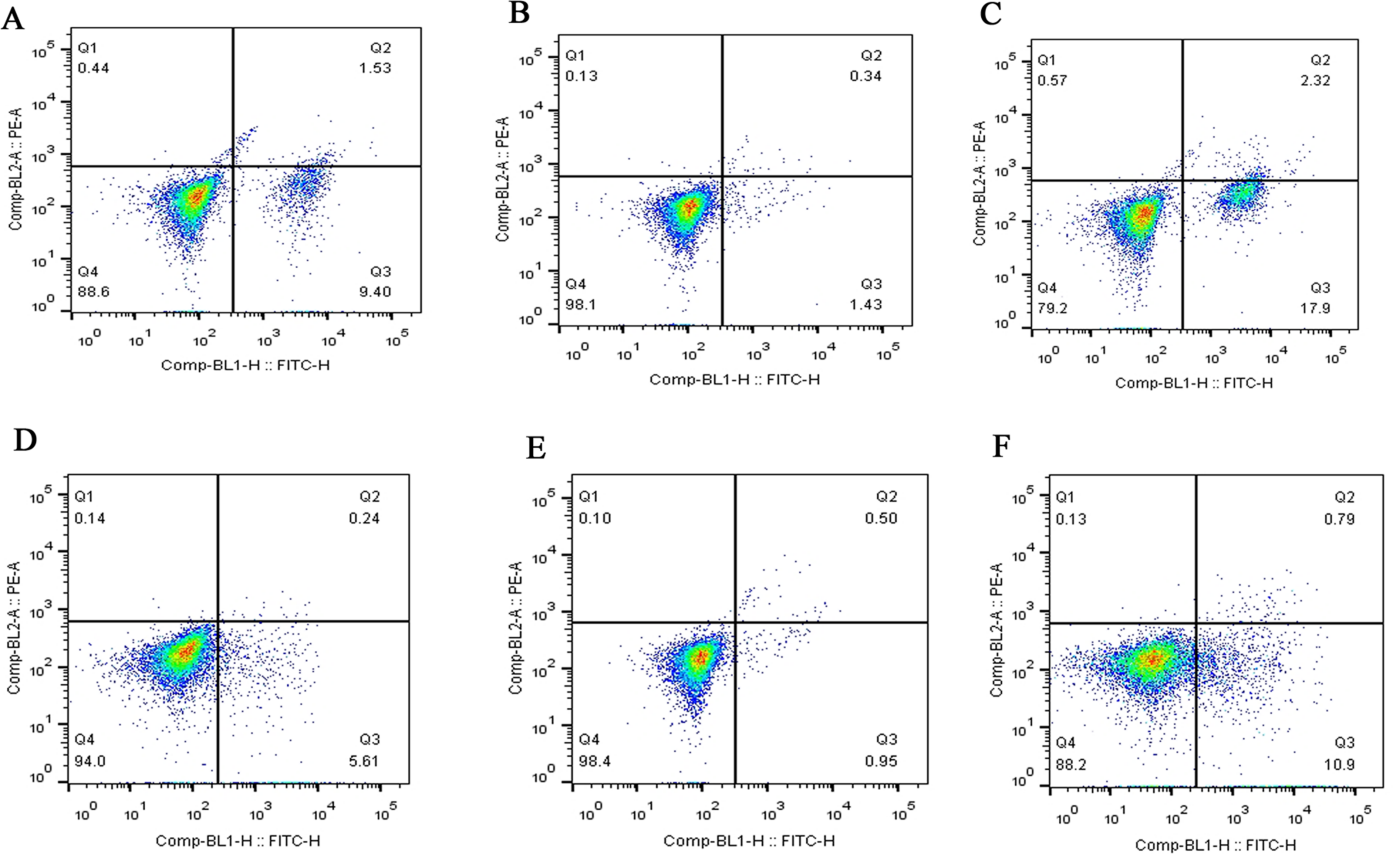
Flow cytometry assay of FLSs following MCP-1 transfection. (A) Normal human FLSs. (B) Overexpression of MCP-1 in human FLSs. (C) MCP-1-silencing in human FLSs. (D) Normal rat FLSs. (E) Overexpression of MCP-1 in rat FLSs. (F) MCP-1-silencing in rat FLSs. The ordinate represents the relative fluorescence intensity. The Q1, Q2, Q3 and Q4 quadrants represents the proportion of necrotic, late apoptotic, early apoptotic and live cells, respectively. MCP-1, monocyte chemoattractant protein-1; FLSs, fibroblast-like synoviocytes.

Flow cytometry was used to detect the effect of MCP-1 on the apoptosis of FLSs. The apoptotic ability of FLSs was weakened by MCP-1 overexpression. On the contrary, the apoptotic ability of FLSs was enhanced when MCP-1 was silenced (Fig. 4). Quantitative analysis of the apoptotic rate of early apoptotic cells (Q3 quadrant) showed that the apoptotic ability of MCP-1 overexpressing cells was weakened in FLSs (Fig. S2).

### Western blotting

**Figure 5:**
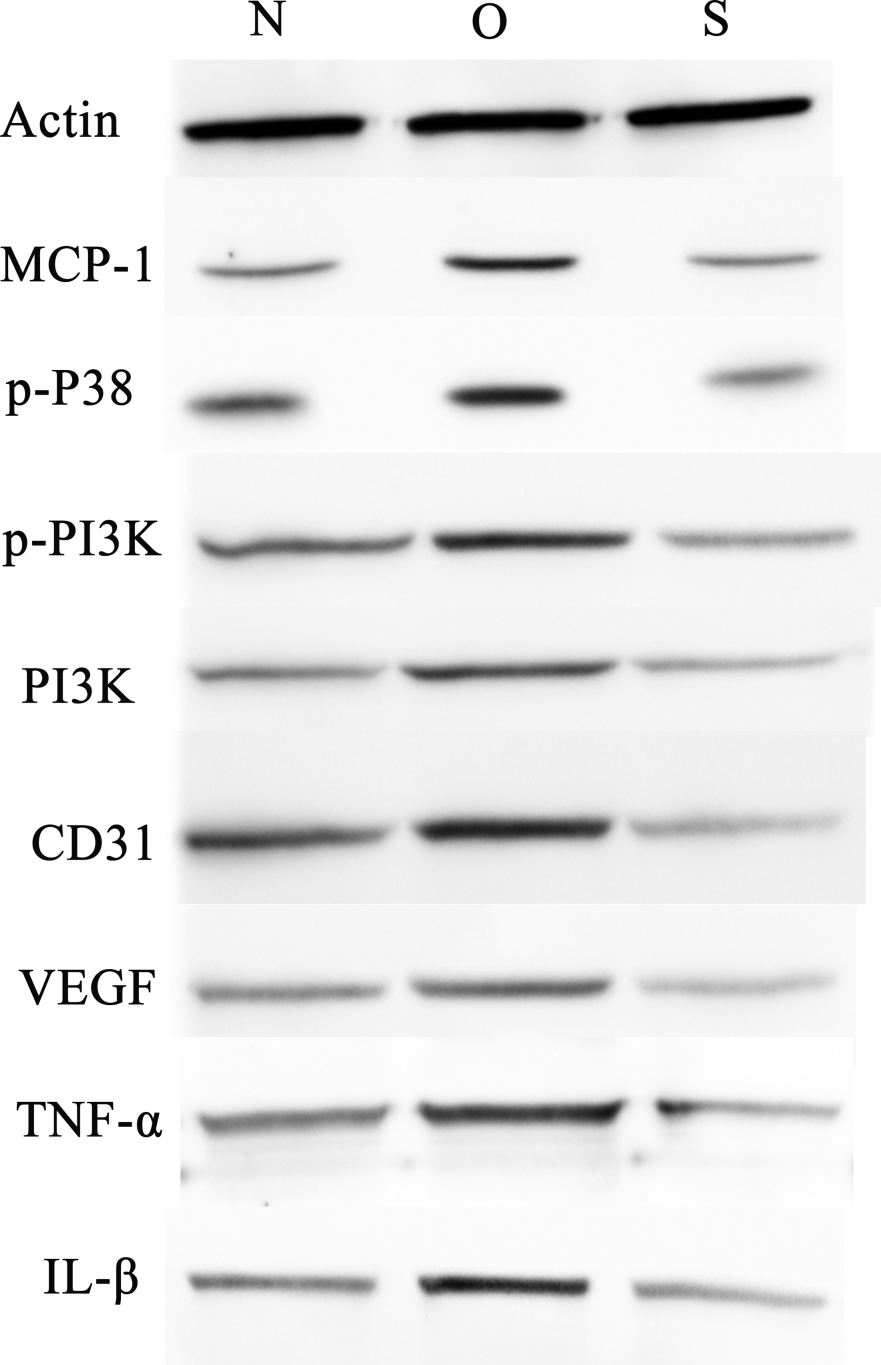
Western blotting assay of MCP-1, p-p38, p-PI3K, PI3K, CD31, VEGF, TNF-α, IL-β following MCP-1 transfection in human FLSs. N, O and S represents the normal control, MCP-1-overexpression and MCP-1-silencing groups, respectively. FLSs, fibroblast-like synoviocytes. Each experiment was repeated for three times.

**Figure 6:**
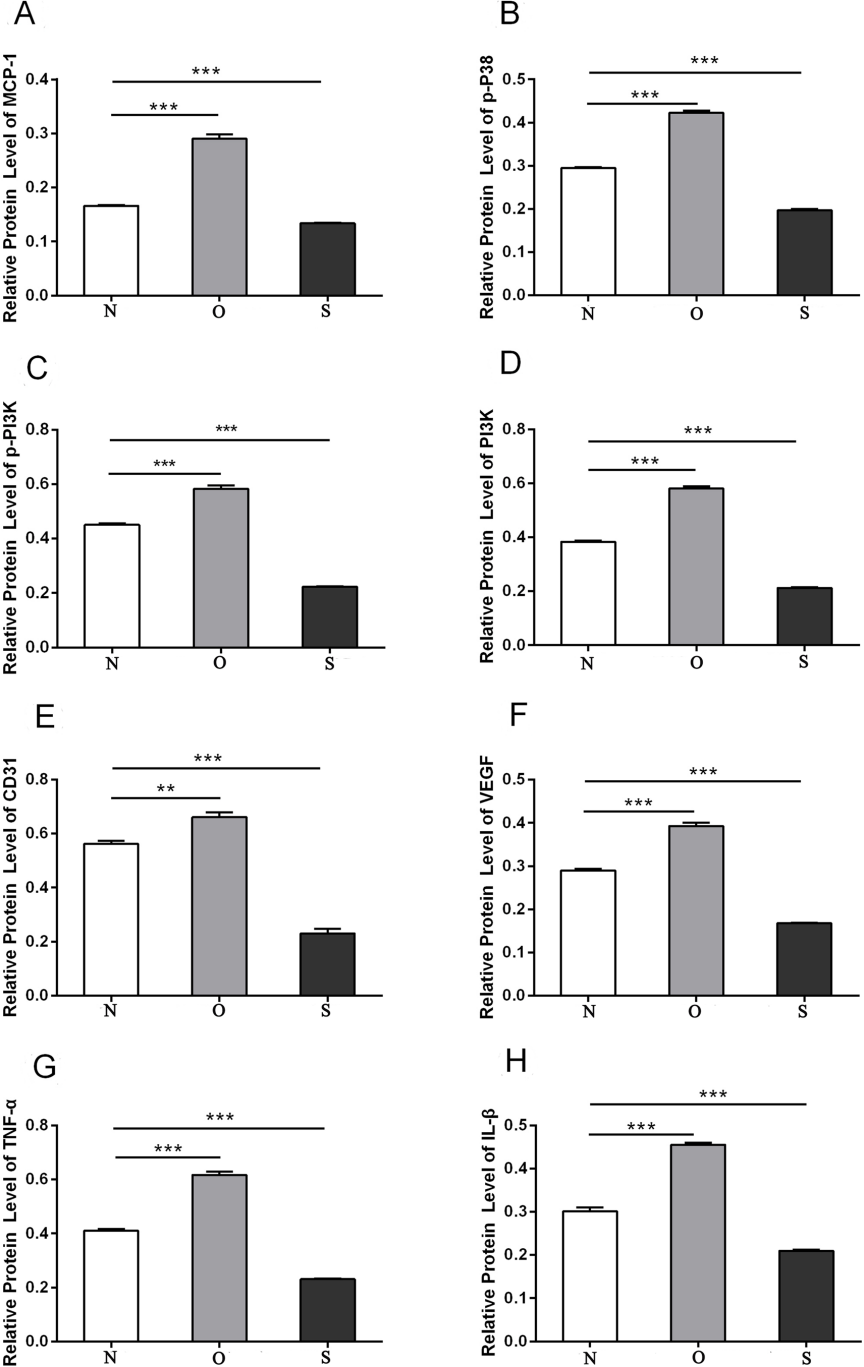
Quantitative analysis of MCP-1 (A), p-p38 (B), p-PI3K (C), PI3K (D), CD31 (E), VEGF (F), TNF-α (G) and IL-β (H) protein bands following MCP-1 transfection in human FLSs. * *P* < 0.05, ** *P* < 0.01 and *** *P* < 0.001. N, O and S represents the normal control, MCP-1-overexpression and MCP-1-silencing groups, respectively. FLSs, fibroblast-like synoviocytes. Each experiment was repeated for three times.

**Figure 7:**
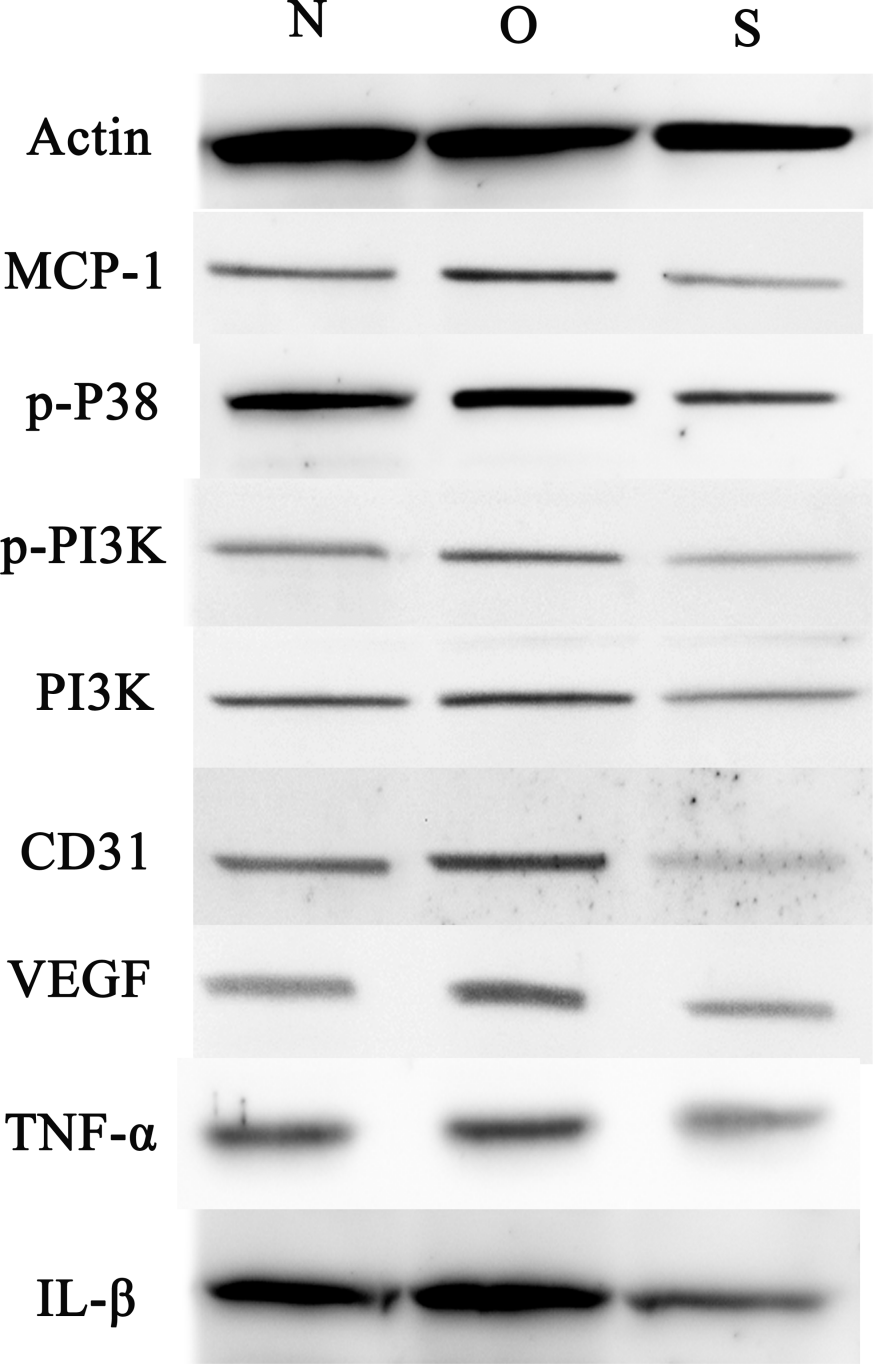
Western blotting assay of MCP-1, p-p38, p-PI3K, PI3K, CD31, VEGF, TNF-α, IL-β following MCP-1 transfection in rat FLSs. N, O and S represents the normal control, MCP-1-overexpression and MCP-1-silencing groups, respectively. FLSs, fibroblast-like synoviocytes. Each experiment was repeated for three times.

**Figure 8:**
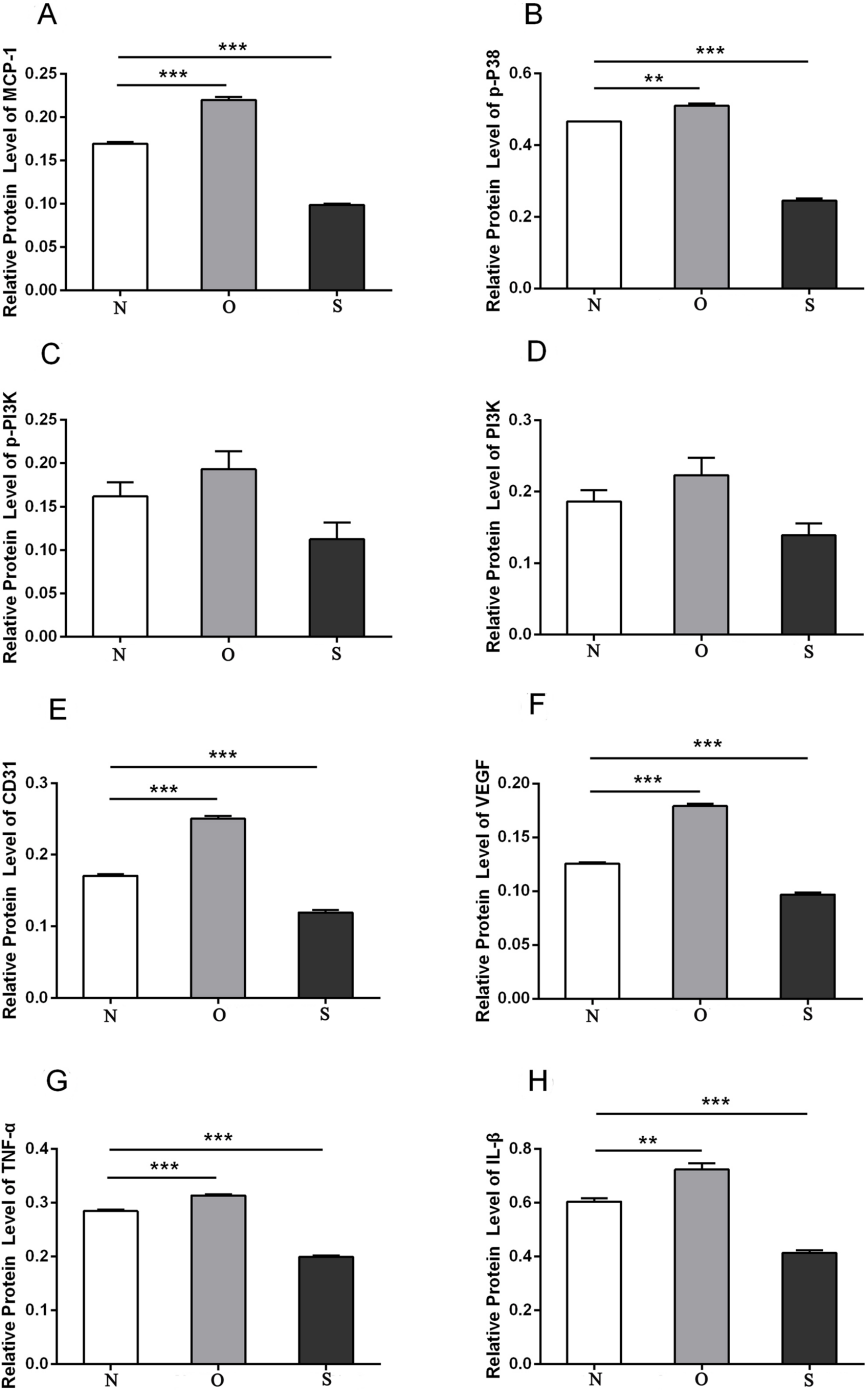
Quantitative analysis of MCP-1 (A), p-p38 (B), p-PI3K (C), PI3K (D), CD31 (E), VEGF (F), TNF-α (G) and IL-β (H) protein bands following MCP-1 transfection in rat FLSs. * *P* < 0.05, ** *P* < 0.01 and *** *P* < 0.001. N, O and S represents the normal control, MCP-1-overexpression and MCP-1-silencing groups, respectively. FLSs, fibroblast-like synoviocytes. Each experiment was repeated for three times.

P-p38 is an important kinase involved in the regulation of the MAPK signaling pathway (Liu et al., 2019b; Zhou et al., 2019). PI3K and p-PI3K are important kinases involved in the regulation of the PI3K/AKT/mTOR signaling pathway (Liu et al., 2019a). TNF-α and IL-β are typical pro- inflammatory cytokines (Charrad et al., 2016). In addition, CD31 and VEGF are members of the immunoglobulin superfamily and VEGF family, respectively (Melinte et al., 2012). Western blotting was used to detect the expression levels of MCP-1, p-P38, p-PI3K, PI3K, CD31, VEGF, TNF-α and IL-β following MCP-1 transfection. In human FLSs, the overexpression of MCP-1 stimulated the expression of p-p38 and p-PI3K, PI3K, CD31, VEGF, TNF-α and IL-1β. The opposite phenomenon was observed in the MCP-1-silencing group (Figs. 5 and 6). In the rat FLSs, following MCP-1 overexpression and silencing, the p-PI3K and PI3K proteins exhibited increased and decreased trends, respectively, albeit not significant. The expression level of other proteins was consistent with that in human FLSs (Figs. 7 and 8).

### MCP-1 is up-regulated in patients with clinical RA

**Figure 9:**
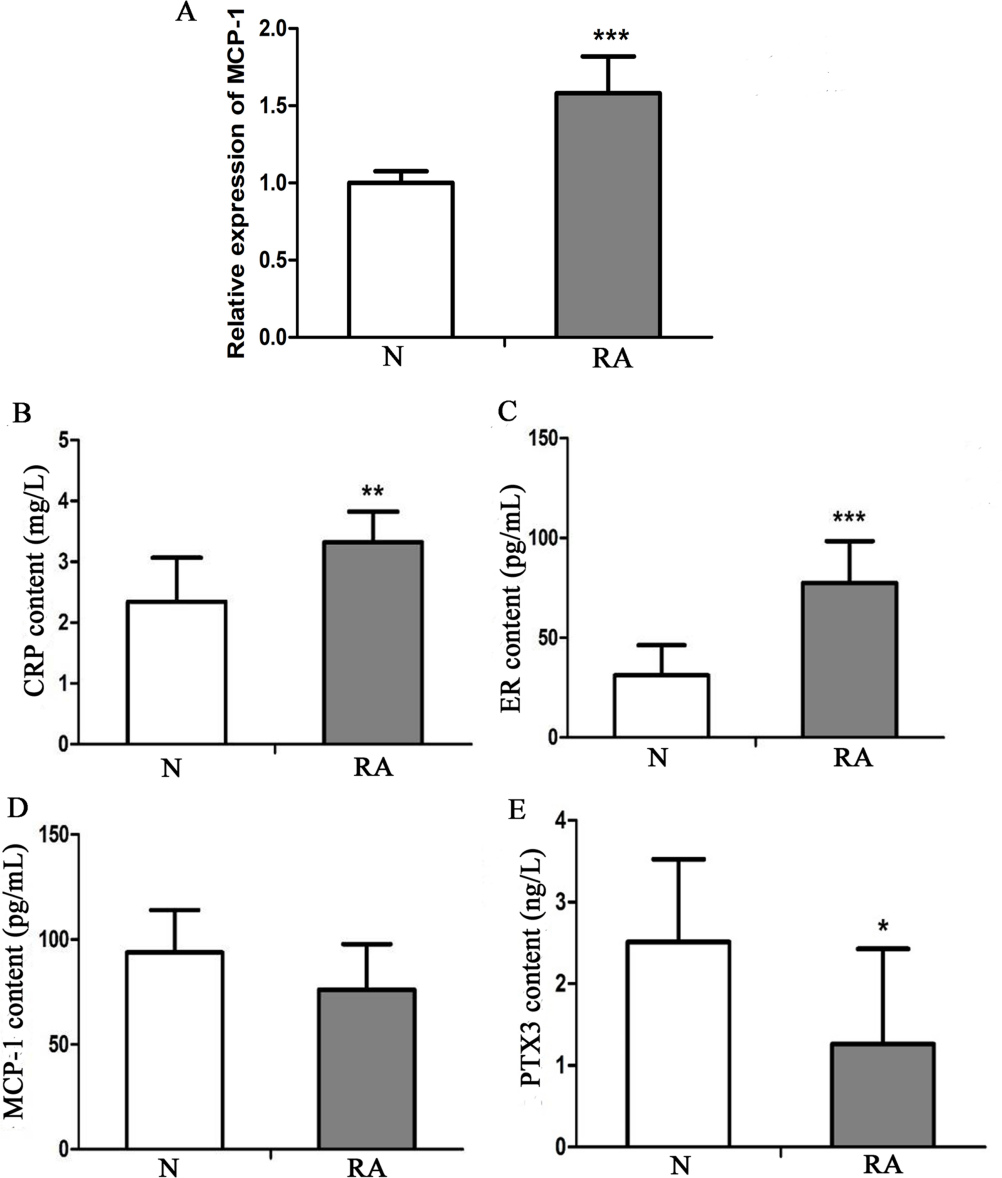
*In vitro* validation of clinical samples. (A) Reverse transcription-quantitative PCR validation of MCP-1 in the blood of patients with RA. (B–E) ELISAs were performed to detect the content of CRP, ER, MCP-1 and PTX3 in the serum of patients with RA. N and RA represents the normal control and patient groups, respectively. ^∗^*P* < 0.05, ^∗∗^*P* < 0.01 and ^∗∗∗^*P* < 0.001. MCP-1, monocyte chemoattractant protein-1; RA, rheumatoid arthritis; CRP, C-reactive protein; ER, estrogen receptor; PTX3, pentraxin-3. Each experiment was repeated for three times.

To investigate the expression level of MCP-1 in clinical RA, 13 patients with RA and 13 healthy individuals were enrolled in the present study. The clinical information of these individuals is presented in Table S1. Blood and serum samples of these individuals were collected for RT-qPCR and ELISA analysis, respectively. In RT-qPCR verification, the mRNA expression level of MCP-1 in patients with RA was increased, as compared with that in healthy individuals, which was consistent with previous studies (Fig. 9A) (Koch et al., 1992; Trzybulska et al., 2018). CRP, ER, MCP-1 and PTX3 are commonly used indicators of patient infection. In patients with RA, the protein levels of CRP and ER was increased, as compared with that in healthy individuals (Figs. 9B and 9C). However, the protein level of MCP-1 in patients with RA was not obvious change, as compared with that in healthy individuals (Fig. 9D). The protein level of PTX3 in patients with RA was decreased, as compared with that in healthy individuals (Fig. 9E). CRP, ER, MCP-1 and PTX3 may play a significant regulatory role in improving arthritis.

### Correlation analysis of clinical data

**Figure 10:**
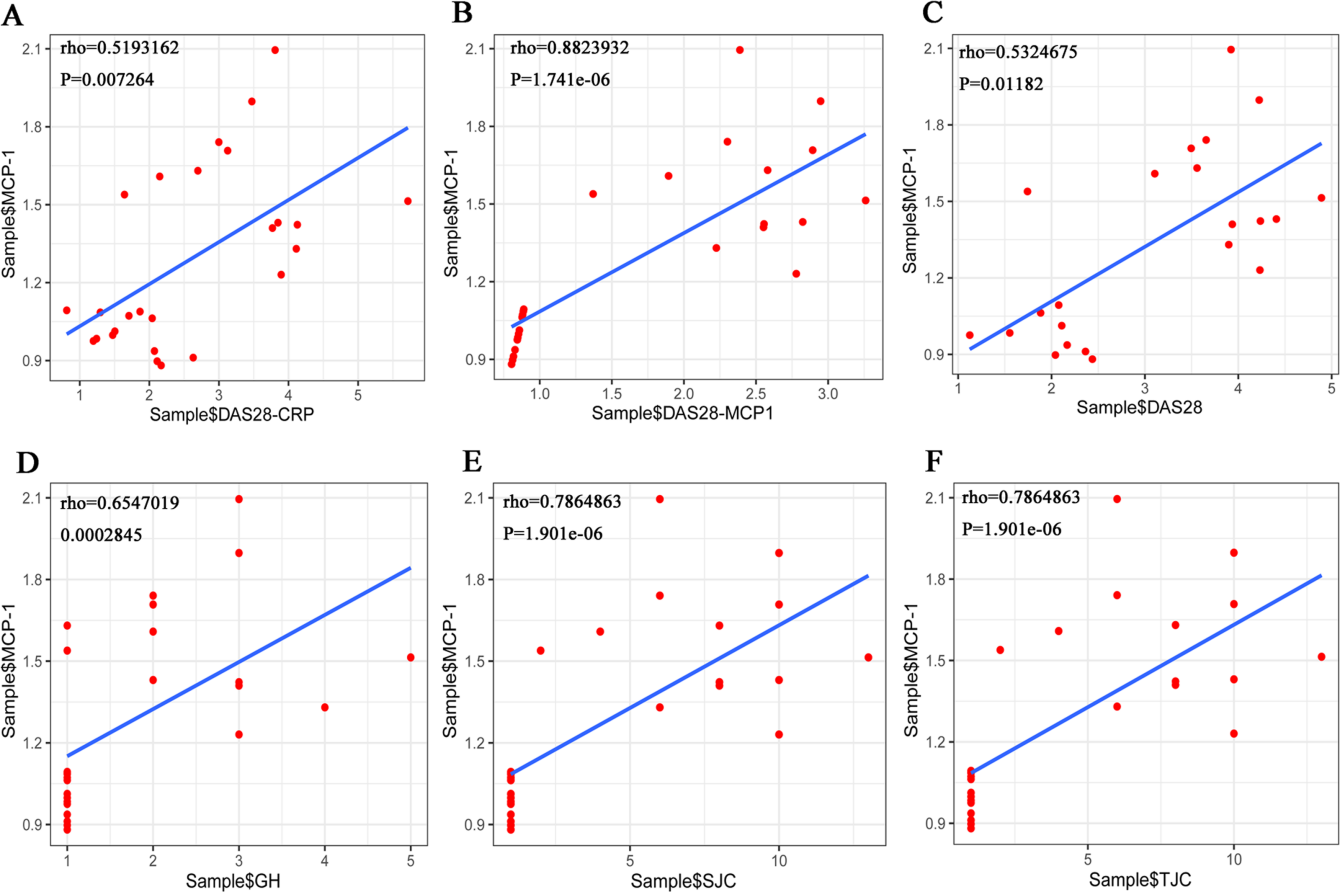
(A–F) Overall correlation analysis between MCP-1 and GH, SJC, TJC, DAS28-CRP, DAS28-MCP-1 and DAS28 expression. Rho, correlation; MCP-1, monocyte chemoattractant protein-1; GH, general health; SJC, swollen joint count; TJC, tender joint count; CRP, C-reactive protein.

The Shapiro–Wilk Test showed that the data on TJC, SJC, GH and MCP-1, among others, in the RA samples were normally distributed. However, when the total sample was tested, the distribution was not normal. Pearson’s correlation analysis was performed to evaluate the association between MCP-1 expression and TJC, SJC, GH in patients with RA. The results showed that there was no correlation between the three indicators (TJC, SJC and GH) and MCP-1. The Spearman’s correlation coefficient was used for the total sample. The results showed that MCP-1 was strongly correlated with each score (Fig. 10). MCP-1 was strongly correlated with TJC, SJC, GH and DAS28-MCP-1 (Figs. 10B, 10D, 10E and 10F) and moderately correlated with DAS28 and DAS28-CRP (Figs. 10A and 10C).

### Expression validation, diagnostic and PPI network analysis of MCP-1

The expression verification results showed that compared with the control group, the expression level of MCP-1 in RA group had an increased tendency without significant difference (Fig. S3A). In the receiver operating characteristic (ROC) analysis, the area under curve (AUC) of MCP-1 was 0.611 (Fig. S3B). Small sample size in dataset may account for the lack of significant differences and diagnostic value in results. In addition, in order to understand the interaction of MCP-1 with other proteins in RA. A PPI network was constructed (Fig. S3C). The results showed that MCP-1 mostly interacted with inflammatory cytokines.

## Discussion

In the present study, MCP-1 was found to promote the cell proliferation and migration, and inhibit the apoptosis of FLSs. In addition, MCP-1 affected the cell cycle of FLSs. Of note, MCP-1 could promote the expression of p-p38, p-PI3K, PI3K, CD31, VEGF, TNF-α and IL-β. In patients with clinical RA, the expression levels of MCP-1 was higher than that of healthy individuals. PPI analysis showed that MCP-1 mainly interacted with other inflammatory cytokines (IL4, IL6, IL10, IL13, CXCL8, TNF). RA is an inflammatory disease, and MCP-1 is a major pro-inflammatory cytokines (He et al., 2006; Scott, Wolfe & Huizinga, 2010). The experimental results in this article suggested that MCP-1 may play an important role in the formation and development of RA.

MCP-1 can promote the proliferation, migration and differentiation of FLSs (Tong et al., 2020). FLSs are mesenchymal cells in the synovial joint and play an important role in the pathogenesis of RA (Liu et al., 2020). MCP-1 is a potent chemokine involved in RA immune regulation and inflammatory processes (Pavkova Goldbergova et al., 2012). Recent studies have shown that the level of MCP-1 is elevated in the synovial tissue, synovial fluid, and plasma of patients with RA (Ellingsen, Buus & Stengaard-Pedersen, 2001; Pavkova Goldbergova et al., 2012). In addition, the ability of FLSs to secrete MCP-1 is increased in joint damage caused by bacterial infection (Scian et al., 2011). In the present study, the expression of MCP-1 was increased in the blood of patients with clinical RA, which is consistent with previous reports. MCP-1 is an important chemokine that regulates the migration and tissue infiltration of monocytes/macrophages (Karimian et al., 2017; Zhu et al., 2014). Previous studies have shown that MCP-1 can also stimulate the migration, invasion and proliferation of tumor cells (Dutta et al., 2018; Küper, Beck & Neuhofer, 2016). In the present study, the overexpression of MCP-1 promoted the cell proliferation and migration and inhibited the apoptosis of FLSs. However, MCP-1-silencing reversed this effect. Therefore, we hypothesized that MCP-1 could play an important role in the pathogenesis of RA by regulating FLSs.

The expression level of p-p38 is up-regulated in osteoarthritis cartilage or isomycin-induced chondrocytes, which suggested that p-p38 may play a role in the progression of osteoarthritis (Ji et al., 2019; Liu et al., 2019b). We found that MCP-1 could regulate the expression of p-p38. Therefore, it is speculated that MCP-1 may regulate articular cartilage or chondrocytes through p-p38, which could regulate the pathological mechanism of rheumatoid osteoarthritis. The expression levels of PI3K and p-PI3K are reduced when inflammation is inhibited (Feng & Qiu, 2018). In addition, it is found that the inhibition of the PI3K/AKT/mTOR signaling pathway accelerates cell apoptosis and autophagy to reduce the development of RA (Feng & Qiu, 2018; Liang, Li & Gong, 2020; Liu et al., 2019a). We found that MCP-1 could regulate the expression levels of PI3K and p-PI3K. Therefore, it is speculated that MCP-1 might regulate PI3K/AKT/mTOR signaling pathway through PI3K, and thus played an important role in apoptosis and autophagy of FLSs. In addition, MCP-1 could regulate the inflammatory progression of RA by regulating PI3K and p-PI3K. However, the specific molecular mechanism remains to be further research. The expression levels of CD31 and VEGF were increased in the invasion area of RA, which implies that CD31 and VEGF may play an important role in the RA development (Ishikaw et al., 2002; Melinte et al., 2012). We found that MCP-1 could regulate the expression levels of CD31 and VEGF. Therefore, we speculated that MCP-1 could play an important role in the formation and development of RA by regulating CD31 and VEGF to affect the invasion of FLSs cells. Certain pro-inflammatory cytokines, such as TNF-α and IL-1, stimulate inflammation and degradation of bone and cartilage, which play a significant role in the pathogenesis of RA (Alam, Jantan & Bukhari, 2017; Mateen et al., 2016). At present, anti-cytokines appear to be effective drug molecules for the treatment of RA (Alam, Jantan & Bukhari, 2017; Mateen et al., 2016). We found that MCP-1 could regulate the expression of proinflammatory cytokines TNF-α and IL-1. Therefore, we speculated that MCP-1 could regulate the degree of inflammation in RA by regulating proinflammatory cytokines. In brief, it is suggested that MCP-1 may regulate the pathological mechanism of RA from multiple aspects by regulating these proteins.

The deletion of the ERα gene can inhibit the protective effect of E2 on synovitis and joint destruction (Engdahl et al., 2014). In addition, ERβ activation also plays a role in the improvement of arthritis (Yang et al., 2010). The expression level of CRP is elevated and regarded as a biomarker in RA (Atzeni et al., 2017; Wasserman, 2018). In RA, the level of PTX3 has been found to be higher than that in the control group (Balbaloglu & Ozcan, 2020). As compared traditional biomarkers, PTX3 has been found to be a sensitive non-invasive biomarker of clinical arthritis activity in RA (Sharma et al., 2018), indicating that ER, CRP and PTX3 may play a significant role in improving arthritis. In the present study, the expression level of CRP was increased in patients with clinical RA, which was consistent with the findings of previous studies. However, ER and PTX3 had a deviation, which requires additional larger samples for further study.

SJC, TJC, GH and DAS28 are designated as the measurement indicators of clinical arthritis activity (Prevoo et al., 1995). It has been hypothesized that MCP-1 and DAS28-MCP-1 in the blood may be conducive to monitoring RA activity (Liou et al., 2013). In the present study, MCP-1 had a strong correlation with each score in the total sample. This suggested that the adapted MCP-1 is a helpful marker of RA clinical disease activity.

The present study had certain limitations. First, the clinical sample size is too small, leading to a certain degree of error in ELISA analysis. Further research on a larger sample size is required. Second, exogenous MCP-1 needs to be used for further functional validation experiments. Third, the expression levels of migratory proteins, p38, AKT, ERK and c-Jun in the MAPK signaling pathway should be further analyzed. In addition, whether the addition of MCP-1 inhibitors would impede the changes observed in the present study needs to be clarified.

## Conclusion

MCP-1 promoted FLS proliferation and migration and inhibited the apoptosis of FLSs. In addition, overexpression of MCP-1 also promoted the expression of p-p38, p-PI3K, PI3K, CD31, VEGF, TNF- *α* and IL-β. However, MCP-1-silencing reversed this effect. This suggests that MCP-1 promotes the progression of RA inflammation. In patients with clinical RA, the expression level of MCP-1 was increased. Moreover, CRP expression was significantly up-regulated compared with healthy individuals. Clinically, MCP-1 was strongly correlated with tender joint count, swollen joint count, visual analog scale for general health and disease activity score 28 (DAS28)-MCP-1, and was moderately correlated with DAS28 and DAS28-CRP. This further suggested that MCP-1 might play a significant regulatory role in RA, and could be used as a measurement index of clinical arthritis activity.

##  Supplemental Information

10.7717/peerj.11973/supp-1 Supplemental Information 1 Detection of MCP-1 transfection efficiency by reverse transcription-quantitative PCR and immunofluorescence (A) Relative expression levels of MCP-1 following transfection in human FLSs. (B) Relative expression of MCP-1 following transfection in rat FLSs. (C) Normal human FLSs. (D) Overexpression of MCP-1 in human FLSs. (E) MCP-1-silencing in human FLSs. (F) Normal rat FLSs. (G) Overexpression of MCP-1 in rat FLSs. (H) MCP-1-silencing in rat FLSs. N, O and S represent the normal control, MCP-1-overexpression and MCP-1-silencing groups, respectively. ** *P* < 0.01 and *** *P* < 0.001. Magnification, x400. Blue fluorescence was emitted by the nucleus, and green fluorescence by the MCP-1 protein. MCP-1, monocyte chemoattractant protein-1; FLSs, fibroblast-like synoviocytes. Each experiment was repeated for three times.

10.7717/peerj.11973/supp-2 Supplemental Information 2 Quantitative analysis of early apoptotic cells (Q3 quadrant) by flow cytometry Apoptotic rate of (A) human and (B) rat FLSs. N, O and S represent the normal control, MCP-1-overexpression and MCP-1-silencing groups, respectively. MCP-1, monocyte chemoattractant protein-1; FLSs, fibroblast-like synoviocytes.

10.7717/peerj.11973/supp-3 Supplemental Information 3 Expression validation (A), diagnostic analysis (B) and protein-protein interaction (PPI) network analysis (C) N and RA represent the normal control and patient groups, respectively. ROC, Receiver operating characteristic; AUC, area under curve.

10.7717/peerj.11973/supp-4 Supplemental Information 4 Clinical information of patients with RA and normal controls N, normal group; RA, rheumatoid arthritis patient group; WBC, white blood cell count; BPC, blood platelet count; ESR, erythrocyte sedimentation rate; CRP, C-reactive protein; SJC, swollen joint count; TJC, tender joint count; DAS28, disease activity score 28; GH, visual analog scale for general health; MCP-1, Monocyte chemoattractant protein-1; NA, not available

10.7717/peerj.11973/supp-5 Supplemental Information 5 ARRIVE 2.0 Checklist

10.7717/peerj.11973/supp-6 Supplemental Information 6 Raw numerical data for Fig. 2

10.7717/peerj.11973/supp-7 Supplemental Information 7 Raw numerical data for Fig. 6

10.7717/peerj.11973/supp-8 Supplemental Information 8 Raw numerical data for Fig. 8

10.7717/peerj.11973/supp-9 Supplemental Information 9 Raw numerical data for Fig. 9

10.7717/peerj.11973/supp-10 Supplemental Information 10 Raw numerical data for Fig. 10

10.7717/peerj.11973/supp-11 Supplemental Information 11 Full-length uncropped blots

## Additional Information and Declarations

### Data Availability

The following information was supplied regarding data availability: Raw data are available as Supplemental Files.
