# Supplementary figures and images for: Exploring the role of monocyte chemoattractant protein-1 in fibroblast-like synovial cells in rheumatoid arthritis
Source: PeerJ. 2021 Aug 11;9:e11973. doi: 10.7717/peerj.11973 (PMC8364321; doi:10.7717/peerj.11973)

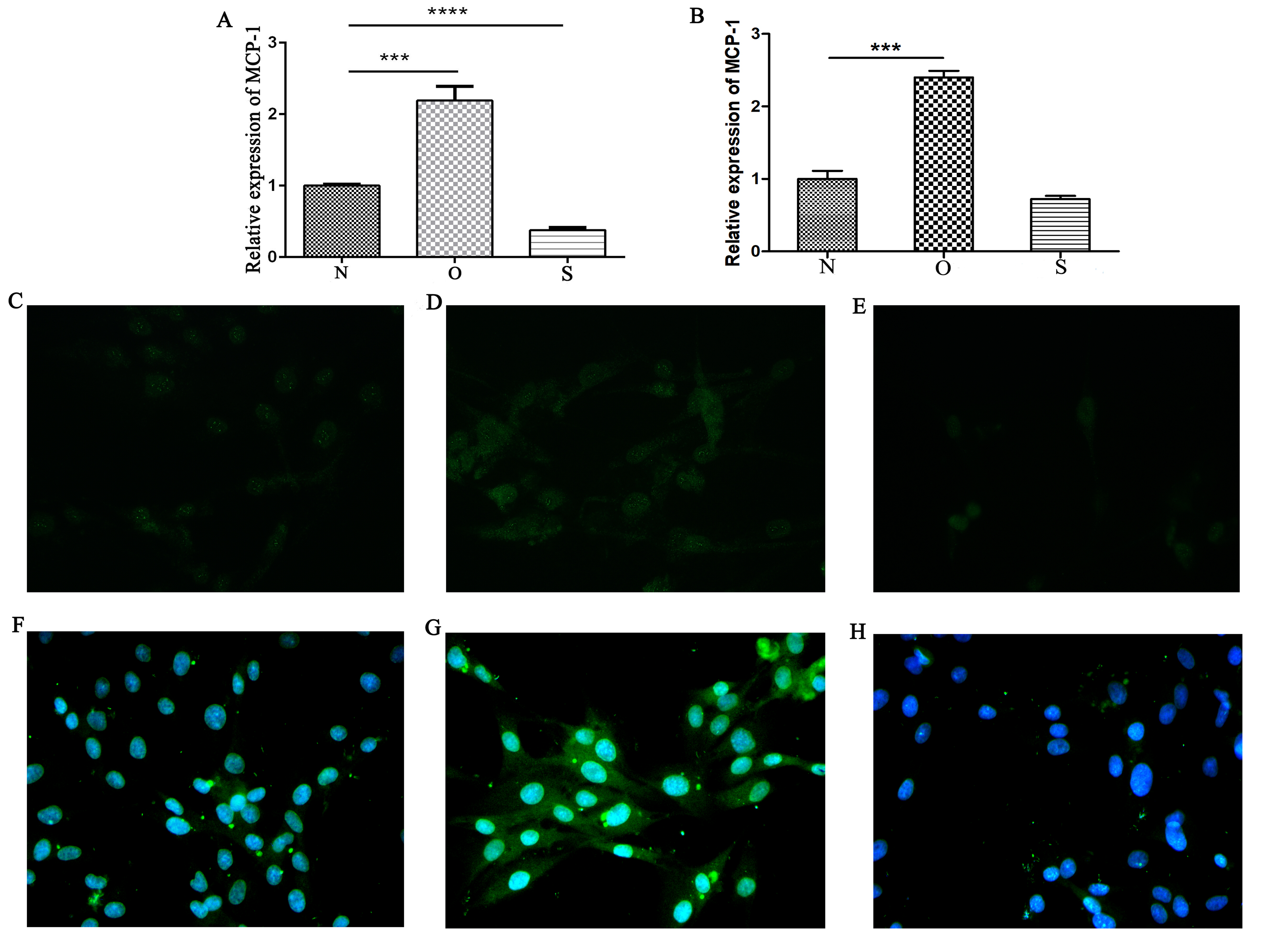

Supplement: Supplemental Information 1 — (A) Relative expression levels of MCP-1 following transfection in human FLSs. (B) Relative expression of MCP-1 following transfection in rat FLSs. (C) Normal human FLSs. (D) Overexpression of MCP-1 in human FLSs. (E) MCP-1-silencing in human FLSs. (F) Normal rat FLSs. (G) Overexpression of MCP-1 in rat FLSs. (H) MCP-1-silencing in rat FLSs. N, O and S represent the normal control, MCP-1-overexpression and MCP-1-silencing groups, respectively. ** P < 0.01 and *** P < 0.001. Magnification, x400. Blue fluorescence was emitted by the nucleus, and green fluorescence by the MCP-1 protein. MCP-1, monocyte chemoattractant protein-1; FLSs, fibroblast-like synoviocytes. Each experiment was repeated for three times. [file peerj-09-11973-s001.png]

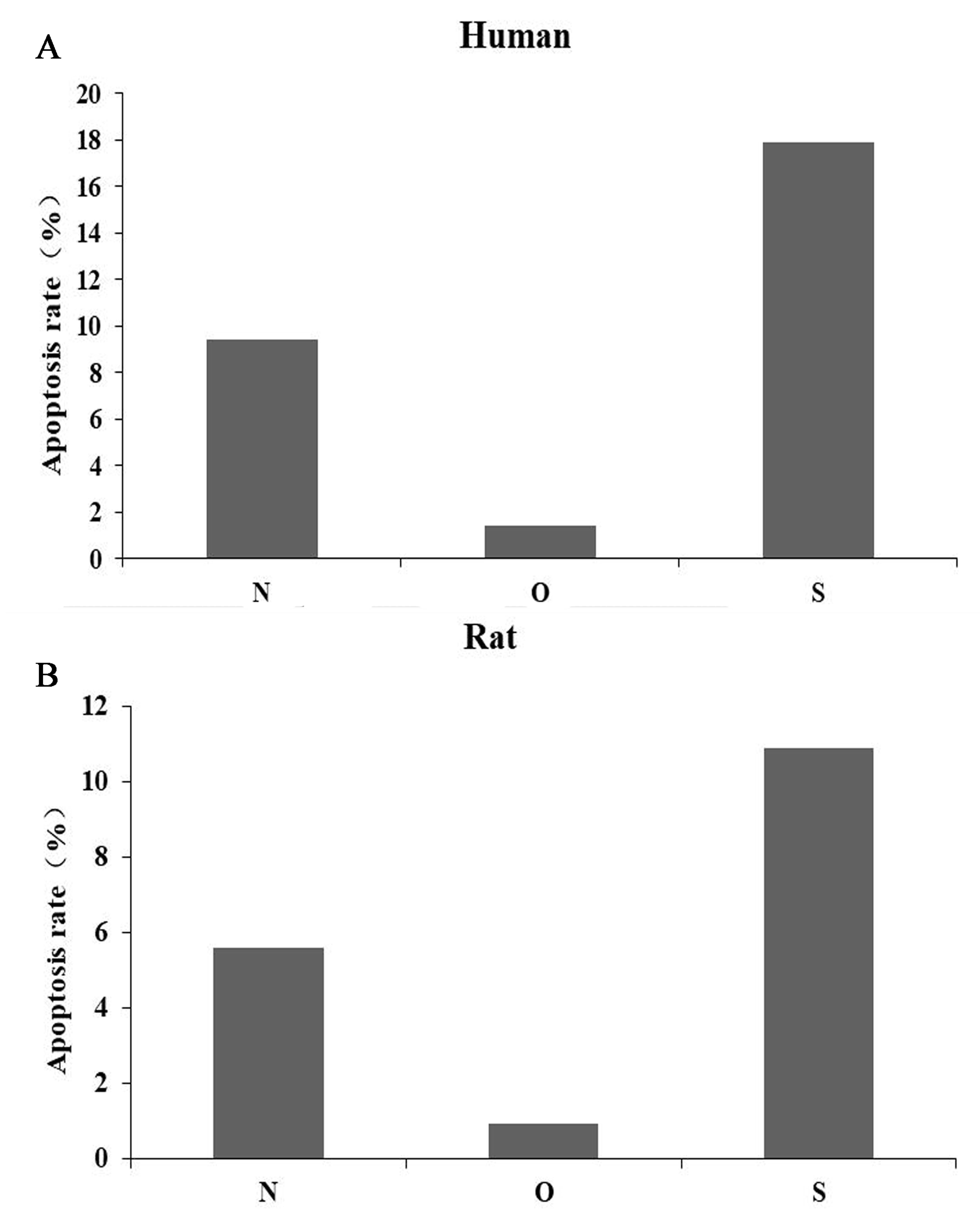

Supplement: Supplemental Information 2 — Apoptotic rate of (A) human and (B) rat FLSs. N, O and S represent the normal control, MCP-1-overexpression and MCP-1-silencing groups, respectively. MCP-1, monocyte chemoattractant protein-1; FLSs, fibroblast-like synoviocytes. [file peerj-09-11973-s002.png]

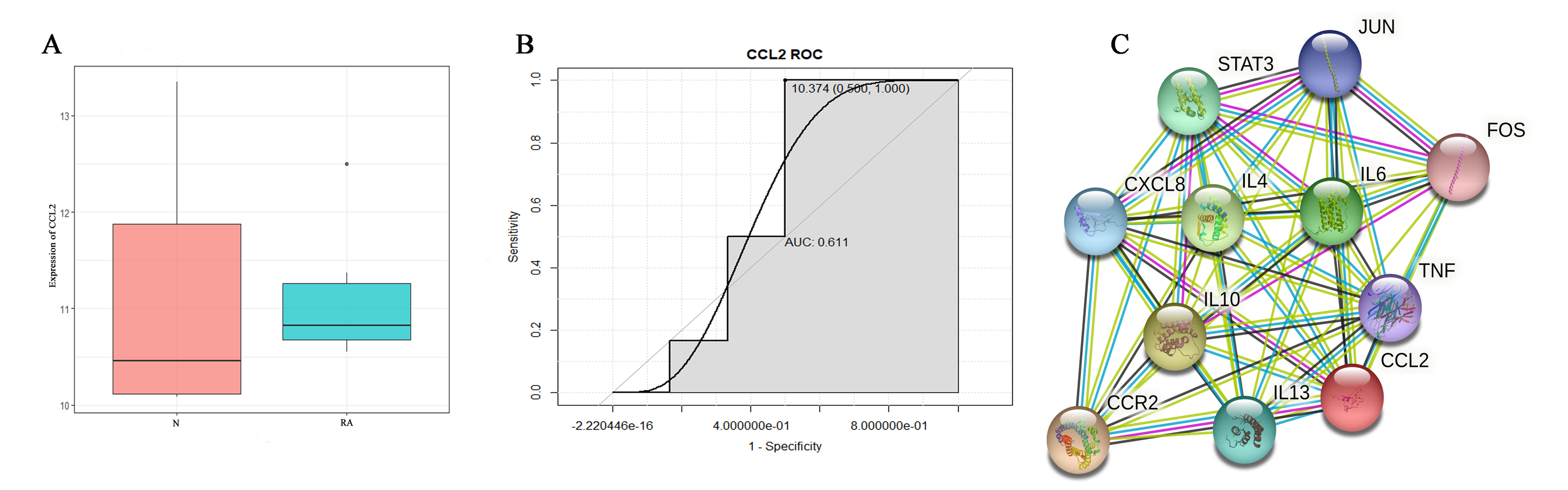

Supplement: Supplemental Information 3 — N and RA represent the normal control and patient groups, respectively. ROC, Receiver operating characteristic; AUC, area under curve. [file peerj-09-11973-s003.png]
