# Supplementary material for: Exploring the role of monocyte chemoattractant protein-1 in fibroblast-like synovial cells in rheumatoid arthritis
Source: PeerJ. 2021 Aug 11;9:e11973. doi: 10.7717/peerj.11973 (PMC8364321; doi:10.7717/peerj.11973)
Supplement: Supplemental Information 4 — N, normal group; RA, rheumatoid arthritis patient group; WBC, white blood cell count; BPC, blood platelet count; ESR, erythrocyte sedimentation rate; CRP, C-reactive protein; SJC, swollen joint count; TJC, tender joint count; DAS28, disease activity score 28; GH, visual analog scale for general health; MCP-1, Monocyte chemoattractant protein-1; NA, not available [file peerj-09-11973-s004.docx]

**Supplementary Table S1 Clinical information of patients with RA and normal controls**

| **Number** | **N or RA** | **Gender** | **Age** | **Duration of disease** | **Symptom** | **Location** | **WBC (cells/mL)** | **BPC (cells/mL)** | **ESR (mm/hour)** | **CRP (mg/L)** | **X-rays** | **Medication history** | **SJC** | **TJC** | **DAS28** | **GH** | **MCP-1** | **DAS28-MCP1** | **DAS28-CRP** |
| --- | --- | --- | --- | --- | --- | --- | --- | --- | --- | --- | --- | --- | --- | --- | --- | --- | --- | --- | --- |
| 1 | RA | Male | 79 | 10 months | Joint pain and fever | Hands | 6 | 189 | 51 | 21.5 | Hands | Hormone | 10 | 10 | 4.231725 | 3 | 1.23067 | 2.779261 | 3.894854 |
| 2 | RA | Female | 47 | 8 years | Both elbow, wrist, metacarpophalangeal joint, proximal interphalangeal joint burst pain | Many joints | 8.3 | 517 | 99 | 821 | No | No | 13 | 13 | 4.89076 | 5 | 1.513797 | 3.260365 | 5.715767 |
| 3 | RA | Male | 58 | 2 months | Multiple joint swelling and pain | Many joints | 9.2 | 477 | 97 | 168 | Hands and knees | Hormone and nsads | 6 | 6 | 3.897709 | 4 | 1.330283 | 2.224874 | 4.111917 |
| 4 | RA | Female | 74 | 3 years | Multiple joint swelling and pain | Many joints | 3.9 | 109 | 8 | 3.1 | Hands and knees | Hormone | 10 | 10 | 3.495295 | 2 | 1.708048 | 2.8931 | 3.12556 |
| 5 | RA | Male | 60 | 7 months | Polyarticular pain | Many joints | 13.6 | 343 | 83 | 19.8 | No | Methotrexate | 10 | 10 | 4.407661 | 2 | 1.430765 | 2.824015 | 3.848729 |
| 6 | RA | Female | 49 | 5 months | Recurrent multiple joint pain | Many joints | 7.5 | 236 | 20 | 2.2 | No | Hormone | 8 | 8 | 3.558214 | 1 | 1.630808 | 2.580618 | 2.697377 |
| 7 | RA | Female | 51 | 7 years | Polyarticular pain | Many joints | 8.5 | 584 | 107 | 79.8 | Hands and knees | No | 6 | 6 | 3.921975 | 3 | 2.095251 | 2.388044 | 3.807586 |
| 8 | RA | Female | 56 | More then 10 years | Polyarticular pain | Many joints | 4.7 | 267 | 36 | 3.1 | Knees | Hormone | 4 | 4 | 3.105572 | 2 | 1.608648 | 1.893404 | 2.149247 |
| 9 | RA | Female | 75 | 1 year | Polyarticular pain | Many joints | 3.4 | 264 | 106 | 80.1 | No | No | 8 | 8 | 4.23662 | 3 | 1.422957 | 2.555446 | 4.127356 |
| 10 | RA | Female | 41 | 3 years | Polyarticular pain | Many joints | 7.3 | 154 | 4 | 3.1 | No | Hormone | 2 | 2 | 1.742594 | 1 | 1.538885 | 1.370052 | 1.643186 |
| 11 | RA | Male | 65 | 4 years | Polyarticular pain | Many joints | 5.2 | 256 | 56 | 10.4 | No | Methotrexate | 6 | 6 | 3.655459 | 2 | 1.740883 | 2.301785 | 2.998876 |
| 12 | RA | Female | 55 | 16 years | Polyarticular pain | Many joints | 6.7 | 190 | 50 | 7.3 | No | Methotrexate, hydroxychloroquine and hormone | 10 | 10 | 4.224002 | 3 | 1.897436 | 2.94811 | 3.473584 |
| 13 | RA | Male | 66 | 1 mouth | Polyarticular pain | Many joints | 5.1 | 273 | 49 | 32 | Hands and knees | Nonsteroidal anti - inflammatory drug | 8 | 8 | 3.935689 | 3 | 1.410199 | 2.551934 | 3.769516 |
| 1 | N | Male | 58 |  |  |  | 6.8 | 228 | Negative | 4.9 | Hands and knees |  | 1 | 1 | NA | 1 | 0.998152 | 0.853279 | 1.473802 |
| 2 | N | Male | 64 |  |  |  | 4.2 | 124 | 29 | 22.8 | Hands and knees |  | 1 | 1 | 2.167245 | 1 | 0.93676 | 0.828522 | 2.073437 |
| 3 | N | Male | 15 |  |  |  | 6.6 | 316 | Negative | 13.4 | Hands and knees |  | 1 | 1 | NA | 1 | 1.088527 | 0.887082 | 1.866149 |
| 4 | N | Female | 62 |  |  |  | 9.4 | 244 | Negative | 3.1 | Hands and knees |  | 1 | 1 | NA | 1 | 1.085557 | 0.886016 | 1.295247 |
| 5 | N | Female | 88 |  |  |  | 5.5 | 117 | Negative | 8.9 | Hands and knees |  | 1 | 1 | NA | 1 | 1.072396 | 0.881259 | 1.70656 |
| 6 | N | Male | 60 |  |  |  | 6.6 | 186 | 21 | 25.2 | Hands and knees |  | 1 | 1 | 2.041364 | 1 | 0.897533 | 0.811839 | 2.112469 |
| 7 | N | Female | 53 |  |  |  | 11.8 | 264 | 6 | 2.7 | Hands and knees |  | 1 | 1 | 1.552786 | 1 | 0.98416 | 0.847773 | 1.241368 |
| 8 | N | Male | 56 |  |  |  | 11.1 | 179 | 48 | 95.1 | Hands and knees |  | 1 | 1 | 2.363768 | 1 | 0.911366 | 0.817804 | 2.630422 |
| 9 | N | Male | 44 |  |  |  | 4.7 | 218 | 2 | 2.4 | Hands and knees |  | 1 | 1 | 1.124327 | 1 | 0.97549 | 0.844322 | 1.195433 |
| 10 | N | Female | 81 |  |  |  | 7.3 | 244 | 23 | 0.9 | Hands and knees |  | 1 | 1 | 2.076843 | 1 | 1.093428 | 0.888834 | 0.812909 |
| 11 | N | Female | 53 |  |  |  | 4.9 | 131 | 14 | 21 | Hands and knees |  | 1 | 1 | 1.883232 | 1 | 1.062727 | 0.877727 | 2.041364 |
| 12 | N | Male | 52 |  |  |  | 9.2 | 450 | 58 | 29.2 | Hands and knees |  | 1 | 1 | 2.437573 | 1 | 0.881325 | 0.804732 | 2.169926 |
| 13 | N | Male | 60 |  |  |  | 6.5 | 239 | 25 | 5.3 | Hands and knees |  | 1 | 1 | 2.109362 | 1 | 1.012579 | 0.858875 | 1.504406 |
| N: normal group; RA: rheumatoid arthritis patient group; WBC: white blood cell count; BPC: blood platelet count; ESR:erythrocyte sedimentation rate; CRP: C-reactive protein; SJC: swollen joint count; TJC: tender joint count; DAS28: disease activity score 28; GH: visual analog scale for general health; MCP-1:Monocyte chemoattractant protein-1; NA: not available | | | | | | | | | | | | | | | | | | | |
